# Iron deficiency in dogs suffering from atopic dermatitis

**DOI:** 10.1186/s12917-024-04350-y

**Published:** 2024-11-06

**Authors:** Carolina Frizzo Ramos, Pavlos G. Doulidis, Nina Polakova, Iwan A. Burgener, Erika Jensen-Jarolim, Giulia Cimarelli, Lucia Panakova, Franziska Roth-Walter

**Affiliations:** 1 Department for Companion Animals and Horses, Clinical Unit of Small Animals Internal Medicine, Dermatology, University of Veterinary Medicine Vienna, Vienna, Austria; 2 Messerli Research Institute, Department of Interdisciplinary Life Sciences, University of Veterinary Medicine Vienna, Veterinärplatz 1, Vienna, A-1210 Austria; 3 Institute of Pathophysiology and Allergy Research, Center of Pathophysiology, Infectiology and Immunology, Medical University of Vienna, Vienna, Austria; 4 Domestication Lab, Department of Interdisciplinary Life Sciences, Konrad Lorenz Institute of Ethology, University of Veterinary Medicine Vienna, Vienna, Austria

## Abstract

**Background:**

Iron-deficiency is associated with increased morbidity and mortality in non-communicable diseases. However, iron parameters are rarely assessed in dogs. Here, we aimed to assess and correlate iron parameters in dogs suffering from Canine Atopic Dermatitis (CAD) compared to non-atopic, healthy dogs.

**Results:**

For this retrospective study, blood values and sera of 34 dogs with confirmed CAD were compared with 94 healthy non-atopic dogs. In our cohort, dogs with CAD had significantly lower mean corpuscular volume (MCV, ) mean corpuscular hemoglobin (MCH) but higher white blood cell counts due to increased levels of circulating neutrophils and monocytes. CAD patients also had elevated total protein and c-reactive protein (CRP), but lower albumin levels compared to our healthy control dogs, indicated low-grade inflammation in the CAD cohort. Spearman correlations associated negatively clinical symptom (CADESI-4/PVAS) with MCV; ceruloplasmin and hepcidin, but positively with serum iron. Only in the CAD-cohort, MCV, CRP and albumin-levels negatively affected serum iron-levels and were positively associated with ceruloplasmin. Linear regression analysis revealed that serum iron-levels in CAD subjects, were positively dependent on hematocrit (packed cell volume, PCV) and albumin, and negatively dependent with white blood cells and neutrophils numbers. In contrast, in the healthy cohort, hepcidin was the sole factor associated with serum iron.

**Conclusions:**

A decreased iron status was associated with a higher symptom burden. Iron homeostasis differed markedly in healthy and atopic dermatitis dogs. CAD patients had depleted iron-stores and presented themselves with subclinical inflammation.

## Background

Canine atopic dermatitis (CAD) is one of the most common skin disorders in veterinary medicine defined as a genetically predisposed inflammatory and pruritic skin disease [1], in which the etiology is poorly understood. In humans, eczema is linked to anemia [2–4], nutritional (minerals and vitamins) and antioxidative deficiencies [5–12], which also many canine studies suggesting that the same nutritional factors may affect the disease course in dogs suffering from CAD [13–24]. Here, we addressed for the first time the iron status in dogs suffering from CAD and compared them with a healthy control cohort.

As the dog standard diet usually is meat-based, iron uptake is generally considered sufficient.

However, in chronic inflammatory conditions dietary iron-uptake is impaired (mucosal block) [25], which indeed was first described in dogs [26] and is a relevant known clinical problem in humans.

Iron deficiency develops slowly over weeks to months [27]. At the beginning body iron stores are depleted along with an accelerated erythropoiesis to sustain the function. After depletion of body iron stores, erythropoiesis and the production of other iron-containing proteins decline resulting in anemia, which represent an extreme form of iron-deficiency [28–30]. Iron-deficiency is complicated due to the fact, that during inflammation also “functional iron-deficiency” exist, in which even despite adequate iron-stores, iron is entrapped within ferritin and not accessible for cellular functions along with the lower bioavailability through the diet. As such, even when hemoglobin-levels and iron markers are in the reference range, due to its dichotomous function, in inflammation (transferrin, ferritin, hepcidin) and nutrition, iron-deficiency may already be present.

Here we assessed iron parameters in dogs with CAD and compared them with a healthy control group. We hypothesized that in dogs affected by CAD, iron parameters are skewed compared to healthy, non-atopic dogs, that may indicate iron- and nutritional deficiencies due to the presence of subclinical inflammation that may impede iron absorption.

## Results

### Characteristics of study group

Two study cohorts were compared, 98 plasma samples of healthy dogs with C-reactive protein (CRP) values below 10 µg/l and 34 serum samples from dogs suffering from CAD. Allometric data and patient characteristic are displayed in Table 1 showing non-significant differences in age and weight, with relatively more companion breeds being presented in the CAD-cohort, while more mix breeds were in the healthy cohort.

Following breeds participated in the study and were classified into following groups in Table 1:

- Companion breeds: French and English Bulldog, Chihuahua, West Highland White Terrrier, Yorkshire Terrier, Pomeranian, Malteser, Bichon Frise, Cocker Spaniel, Whippet, Staffordshire Bullterrier, Havaneser, Poodle, Elo, Petit Brabancon, Shiba Inu.
- Hunting breeds: Labrador, Golden Retriever, Pointer, Beagle, German Shorthair, Vizzla, Rhodesian Ridgeback, Jack Russel Terrier, Münsterländer, Dachshund, Pinsher, Lagotto Romagnolo, Border Terrier, Irish Terrier, Andalusian.
- Herding breeds: German and Australian Shephard, Akita, ChowChow, Corgi, Husky, Rottweiler, Collie, Border Collie, Puli, Samojede.

In the CAD cohort, 17 dogs received systemic or topical treatment (*n* = 17 of 29, 5 missing information), with 9 CAD patients receiving more than one treatment at the time of blood draw. Twelve dogs were on no systemic therapy and in five patients, information on current treatment was missing, but the possible use of glucocorticoids was excluded. From the systemic treatment, ten patients were on Lokivetmab and one on ciclosporin. One patient was on topical glucocorticoid, and one received topical antihistamines. Eleven patients received regular local shampoo treatment with 4% chlorheximidine,

**Table 1:** Dogs‘characteristics

Characteristics	CAD		Healthy dogs		*p*-Value
	*N* = 34		*N* = 94		
	MW	STDEV	MW	STDEV	
Age	4.7	2.4	5.4	2.6	0.151
Weight	19.0	11.2	17.0	7.7	0.259
CADESI	11.21	9.31	n/a	n/a	
PVAS	4.96	2.36	n/a	n/a	
**Medication use**	N	%			
Lokivetmab	10	29.4			
Ciclosporin	1	2.9			
Topical glucorticoids	1	2.9			
Topical antihistamines	1	2.9			
Topical chlorheximidine	11	32.4			
No treatment	12	35.3			
Missing information	5	14.7			
**Gender distribution**	N	%	N	%	
Female	10	29.4	20	21.3	
Male	10	29.4	12	12.8	
female neutered	10	29.4	32	34.0	
male neutered	4	11.8	30	31.9	
**Breed distribution**	N	%	N	%	
Companion breed	13	38.2	18	19.1	
Hunting breed	10	29.4	19	20.2	
Herding breed	5	14.7	19	20.2	

### Decreased red blood cells, but increased white blood cells in CAD patients

**Fig. 1:**
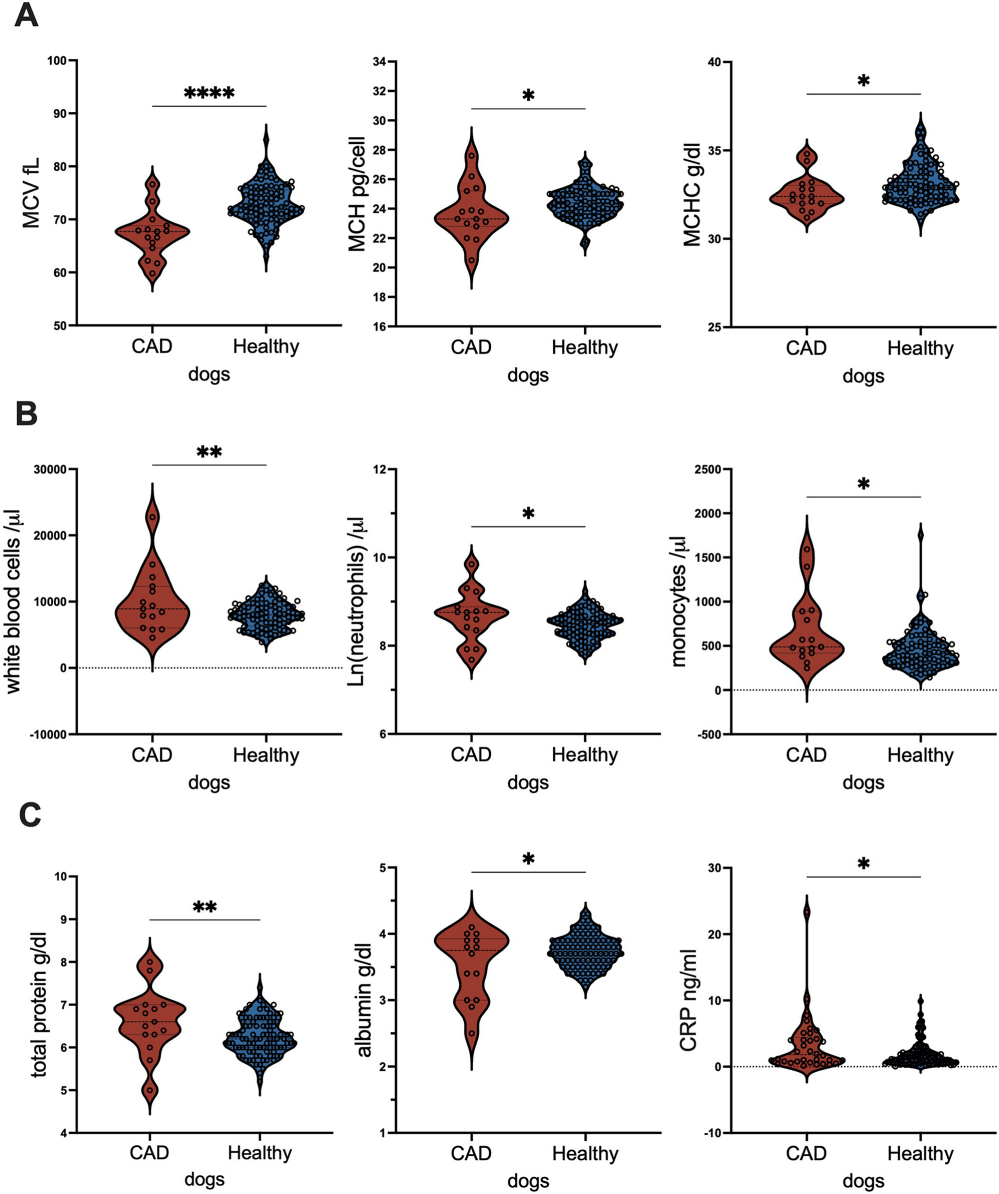
Blood cells and inflammation marker differ in dogs with or without canine atopic dermatitis CAD. Boxplot comparing (**A**) Red blood cell parameters: mean corpuscular volume (MCV) and mean corpuscular hemoglobin (MCH) and mean corpuscular hemoglobin concentration (MCHC); (**B**) White blood cells: white blood cells, total neutrophils and monocyte numbers; and (**C**) Humoral parameters: total protein, albumin and CRP levels in treated dogs with CAD or healthy control dogs. For statistical analyses, groups were tested for normality with Anderson-Darling-test, before parameters were compared with unpaired t-test (MCV MCH, white blood cells, neutrophils, total protein and albumin) or Mann-Whitney U test (monocytes) and Kolmogorov-Smirnov test (CRP). *p* < 0.5, **** *p* < 0.0001

We next compared blood parameters in the CAD and healthy cohort. As depicted in Table 2; Fig. 1, though all blood parameters were within the reference range, CAD patients exhibited significantly lower mean corpuscular volume MCV, mean corpuscular hemoglobin MCH and mean corpuscular hemoglobin concentration MCHC. In contrast, in our CAD patients white blood cells and here particularly total neutrophils and monocytes, but not lymphocytes were significantly elevated compared to the healthy cohort. Our CAD patients had significantly elevated total protein and CRP-levels, but significantly lower albumin levels compared to the healthy cohort.

As such, we conclude that already the red and white blood cell parameter already indicate iron-deficiency in our CAD cohort which is accompanied with increased immune activation.

**Table 2:** Blood parameters in CAD and healthy dogs

Red blood parameters	Reference range	CAD		Healthy dogs		*p*-Value
		*N* = 34		*N* = 94		
		MW	STDEV	MW	STDEV	
Hemoglobin, HGB (g/dl)	14.1–20.1	18.23	2.05	17.88	1.84	0.2785
Packed cell volume, PCV (vol%)	41–58	51.27	5.12	53.02	5.23	0.2551
Mean corpuscular volume, MCV (fl.)	64–76	67.16	4.30	72.78	3.79	**< 0.0001**
Mean corpuscular hemoglobin, MCH (pg)	21–26	23.72	1.79	24.41	1.04	**0.0364**
Mean corpuscular hemoglobin concentration, MCHC (g/dl)	31–34	32.56	0.97	33.15	111	**0.0339**
**White blood parameters**						
WBC /µl	5700–14,200	10,027	4701	7963	1971	**0.004**
Total Neutrophils /µl	2700–9400	6706	4181	4910	1521	**0.0379**
Monocytes /µl	100–1300	663.9	389.7	458.3	227.5	**0.0323**
Lymphocytes /µl	900–4700	2063	926.4	2122	761.2	0.6524
Eosinophils /µl	100–2100	438.3	245.4	486.4	266.6	0.8958
Basophils /µl	0–100	79.53	62.5	87.34	48	0.5263
**Inflammation parameters**						
Total Protein (g/dl)	5.5–7.2	6.61	0.75	6.24	0.43	**0.007**
Albumin (g/dl)	3.2–4.1	3.53	0.50	3.74	0.24	**0.0129**
CRP (µg/l)	0–12	3.30	4.33	1.88	1.87	**0.0478**
**Iron parameters**						
transferrin (µg/l)	n/a	118.8	25.74	125.5	36.21	0.484
ferritin (µg/l)	n/a	6.20	3.39	9.82	11.33	0.4175
hepcidin (µg/l)	n/a	5.59	2.43	9.24	7.01	**< 0.0001**
serum iron (µg/l)	970–2630	2243	1692	1625	1126	**0.011**
ceruloplasmin (AU)	n/a	10.8	5.5	60.9	74.3	**< 0.0001**
total iron binding capacity, TIBC µg/L	2800–4890	2631	1645	2317	1407	0.253
transferrin saturation, %TSAT	27–66	40.6	5.6	35.0	6.7	**< 0.0001**

### Depleted iron-stores and increased dietary iron absorption in CAD patients

**Fig. 2:**
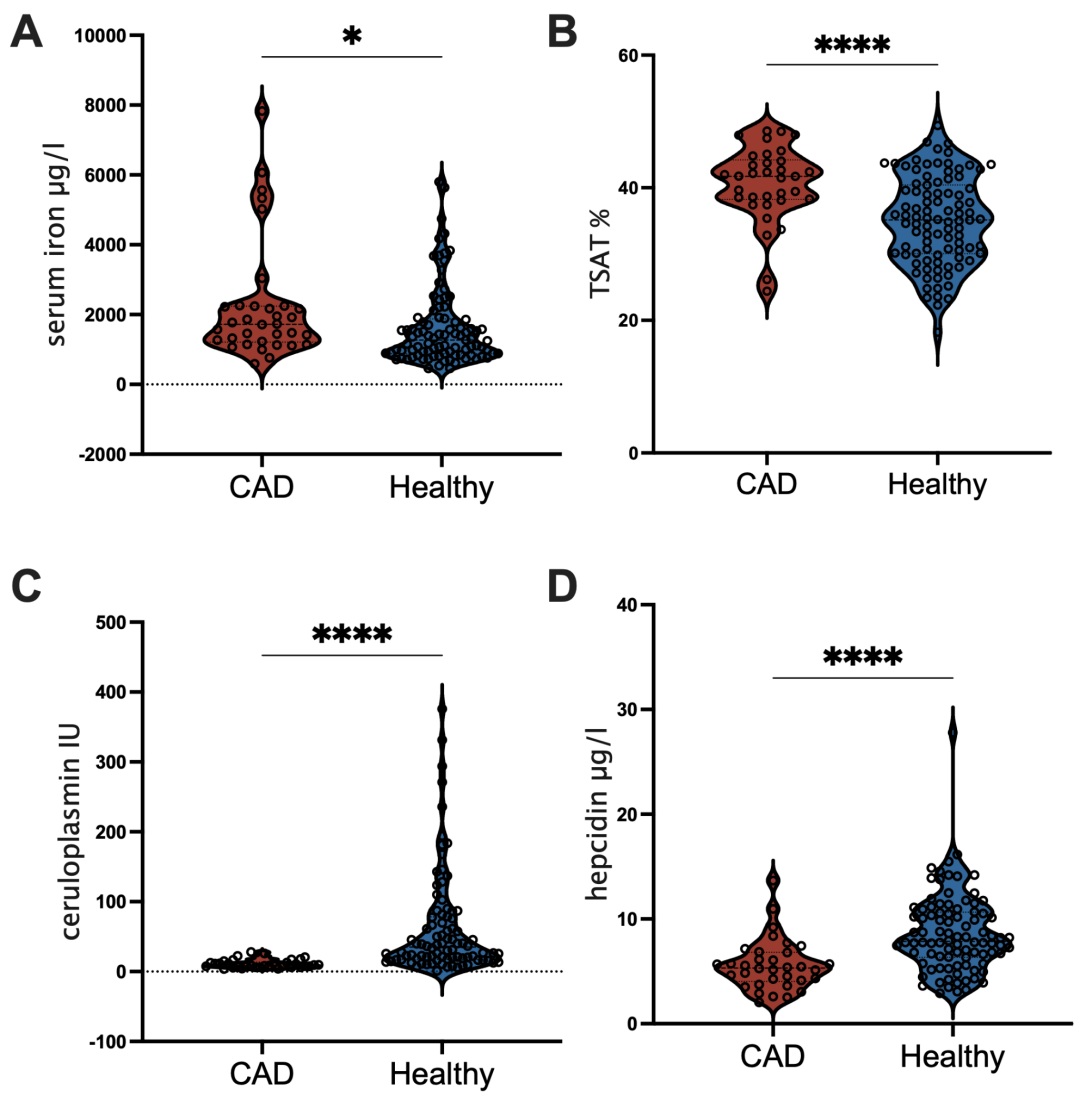
Increased serum **i**ron and TSAT-levels, but lower hepcidin and ceruloplasmin-activity in CAD patients, Comparison of **A**. serum iron, **B**. transferrin saturation TSAT, **C**. ceruloplasmin activity and **D**. hepcidin in dogs with atopic dermatitis (CAD) or not (healthy). Data were assessed for normality, before comparing with t-test for TSAT-levels, or with non-parametric Kolmogorov-Smirnov test for other parameters. ** *p* < 0.01, **** *p* < 0.0001

Next, we compared iron parameters. In our study cohort, dogs with CAD had significantly lower hepcidin- and ceruloplasmin-levels, while – contrary to our expectations – serum iron and the transferrin saturation TSAT, (calculated by measuring total iron binding capacity TIBC) was significantly elevated (Fig. 2).

Thus, our data clearly emphasize that in the study group, CAD patients had depleted iron stores (due to decrease ceruloplasmin-levels) and had very low hepcidin pointing towards the greater dietary iron-demand in dogs with CAD than their healthy counterparts. The observed elevated serum iron levels in the CAD cohort may be explained by a combination of the use of immune-modulating medication, increased hemolysis and the analysis of non-fasting blood.

### Clinical symptoms are associated with depleted iron stores and subclinical inflammation in CAD patients

**Fig. 3:**
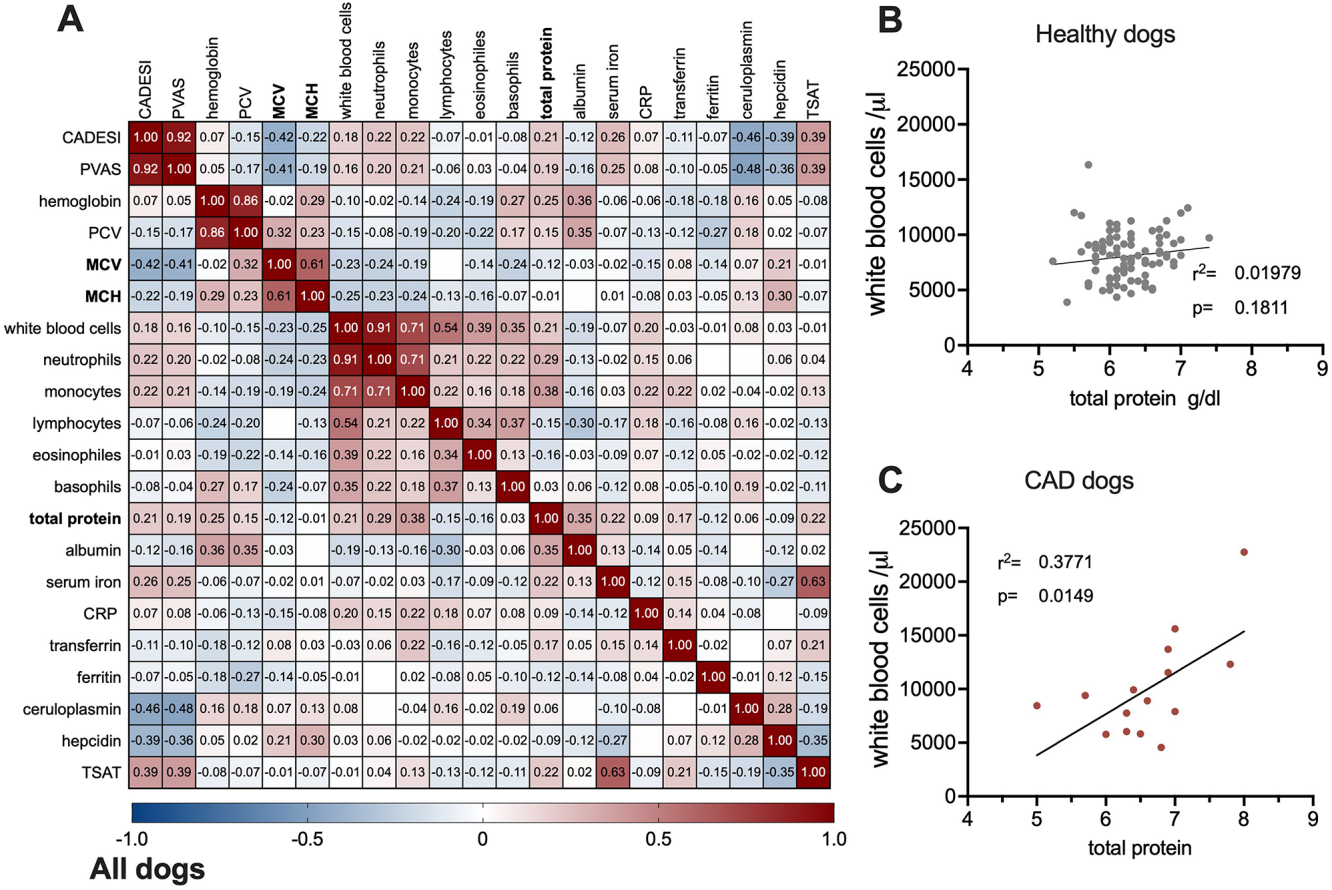
Correlation and regression analysis of clinical symptoms (CADESI/PVAS) in dogs. (**A**). Spearman correlation of CADESI/PVAS with blood parameters with positive association in red hues and negative associations annotated in blue hues. Values below − 0.18 or above 0.18 are significant. Linear regression analyses of total protein (as an indicator of inflammation) with white blood cells in (**B**) healthy (**C**) dogs with CAD. *P* < 0.05 is significant

We next performed a correlation analysis of blood parameters with all dogs to identify possible associations of the clinical symptoms (CADESI-4 and PVAS) with the blood and inflammation-associated parameters. As depicted in Fig. 3A, though a significant association suggested symptoms particularly in dogs with low MCV (Spearman r -0.42, *p* = 0.00001 for CADESI, r -0.41 *p* = 0.00002 for PVAS), ceruloplasmin (r -0.46, *p* = 1 × 10^− 7^ for CADESI, r -0.36 *p* = 2 × 10^− 8^for PVAS), and hepcidin (r -0.39, *p* = 1 × 10^− 5^ for CADESI, r -0.48 *p* = 5 × 10^− 5^for PVAS) and with elevated numbers of neutrophils (r 022/0.20, *p* = 0.023/0.0462 for CADESE/PVAS) and monocytes (r 022/0.20, *p* = 0.023/0.0462 for CADESE/PVAS), regression-analysis did not reveal a direct dependency (data not shown) and being the cause for the symptoms. However, subanalysis of CAD/healthy group again linked inflammation solely to CAD-patients when performing regression analysis. Total protein (as surrogate marker for subclinical inflammation) showed a linear dependency with white blood cell counts only in the CAD cohort, but not in the healthy controls (Fig. 3B and C). Hence, though most of the CAD dogs were on medication (Table 1), subclinical inflammation was still very present in these dogs. Iron-deficiency was associated with symptoms, but we were not able to establish a dependency with the clinical symptoms.

### Iron homeostasis differ in the healthy and CAD cohort

**Fig. 4:**
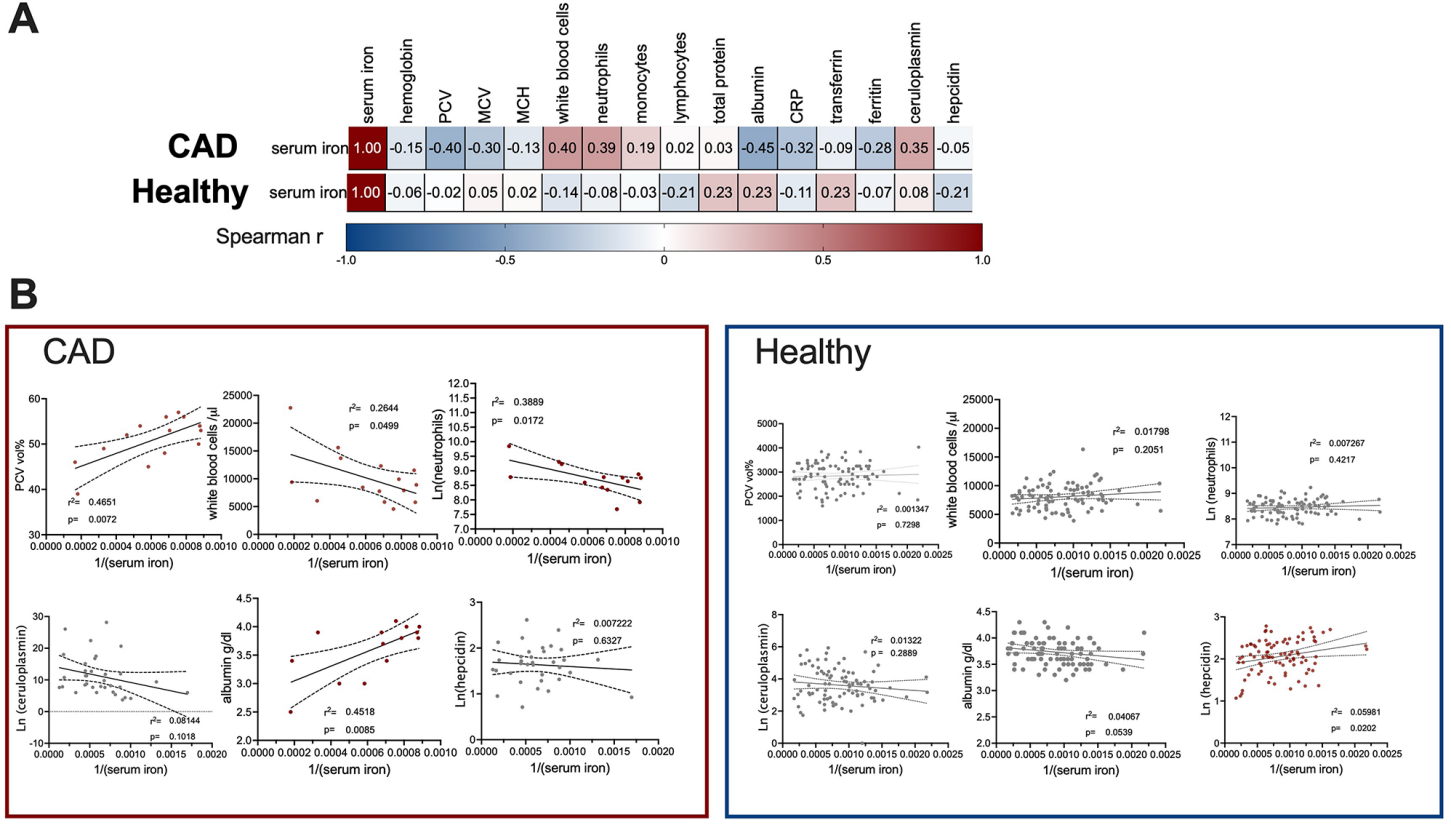
Correlation and regression analyses of serum iron in dogs with CAD or considered healthy. **A**. Spearman correlation of serum iron are in CAD-patients negatively associate with ferritin, MCV, PCV, albumin and CRP, and positive with ceruloplasmin, while in the healthy cohort, serum iron was negatively associated with hepcidin and positive with total protein. **B**. Linear regression analysis (with transformed data to reach parametric distribution) to inversed serum iron showed that serum iron was significantly negatively dependent with PCV and albumin, whereas a positive dependency was seen with white blood cell number and neutrophils in dogs with CAD. In contrast in the healthy cohort, only hepcidin showed a negatively dependency with serum iron-levels. *P* < 0.05 is considered significant

As in our cohort, iron-deficiency was associated, but not directly linked with symptoms, we next sought to investigate factors that influence iron-homeostasis in healthy dogs or suffering from CAD. As depicted in Fig. 4A, the association-profile of serum iron-levels was completely different in CAD versus healthy subjects. CAD serum iron-levels correlated strongly negatively with packed cell volume (PCV) (r-0.45), MCV (r-0.3), albumin (r-0.45), CRP (r-0.32), and was positively associated with white blood cells (r 0.40), total neutrophil (r 0.39), and ceruloplasmin (r 0.35) (Fig. 4B). In contrast, as seen in Fig. 4B in the healthy control group serum iron was negative associated with hepcidin (r-0.21) and lymphocytes(r-0.21) and positively with total protein, transferrin, and albumin (all with r 0.23). A direct significant positive dependency of serum iron-levels with white blood cells and neutrophils and a negative with the PCV and albumin could be established in CAD dogs, whereas in healthy dogs the only parameter directly linked with serum iron-level was hepcidin. As such, we conclude that iron homeostasis differed in healthy vs. CAD dogs. In healthy dogs, dependencies reflected a normal iron homeostasis in an iron replete status. In contrast, in dogs suffering from CAD iron homeostasis were rather linked with inflammation markers and depleted iron stores.

## Discussion

In humans, iron-deficiency is an independent risk factor for all-cause mortality [31] and is associated with an increased morbidity and mortality in a numbers of chronic inflammatory diseases [3, 29, 30, 32–54] including atopic [3, 30, 55] and autoimmune diseases [32, 33] and those affecting the heart [42, 56], the kidneys [38, 39] and the lung [44, 45]. As iron related parameters are infrequently measured in veterinary medicine, this work aimed to measure the iron status in healthy dogs and to compare these with dogs diagnosed with canine atopic dermatitis.

We only found two studies that assessed red blood parameter in dogs with atopic dermatitis and in which dogs probably suffered also from other nutritional deficiencies: One reported severe anemia in their atopic dermatitis patients [57] and another reported significantly lower hemoglobin, but higher neutrophil numbers in their CAD patients [58].

In iron deficiency anemia, red blood cells become smaller and have less hemoglobin. This is because due to a lack of iron, also hemoglobin production is affected. The body aims to compensate the lack of iron by producing more red blood cells (thus increasing cell division) with limiting resources. This results that after each division the red blood cells become smaller [59], with MCV (which measures the average size of red blood cells) being the surrogate marker here. A low MCV that is typically below 60 fL is considered a microcytic trait in dogs and is a hallmark of iron deficiency anemia [60]. As also less hemoglobin is incorporated into the red blood cells, this results in a decrease of the red blood cell parameter MCH and MCHC, which reflect the amount of hemoglobin per red blood cell (MCH) or per volume (MCHC). Low levels of MCH and MCHC due to low hemoglobin concentration is most commonly caused by iron-deficiency. As red blood cells have its vivid color due to hemoglobin binding to iron, (hemoglobin without iron is colorless to yellowish), less iron and/or hemoglobin results in hypochromia with already assessing the color of a blood drop being an inaccurate method for measuring hemoglobin-levels and its incorporated iron [61].

Here, we show that dog patients suffering from CAD had significantly smaller red blood cells, which had significantly lower levels of hemoglobin compared to healthy dogs. The lower red blood cell profile was accompanied by subclinical inflammation in these CAD patients. This was reflected on a cellular level with higher white blood cell counts particularly of neutrophils and monocytes and on the humoral levels with elevated total protein concentration and CRP-values and lower albumin-levels compared to the healthy cohort.

Similar, as in humans where iron-deficiency in apparently healthy individuals goes along with elevated neutrophils and monocyte numbers [62, 63] as well as elevated CRP levels [29, 30, 64], also our CAD cohort depicted higher granulocyte and monocyte counts compared to the healthy control cohort and elevated CRP-levels compared to the healthy control group. In line, total protein, which includes globulin and albumin-levels in the sera, is along the well described positive acute phase protein CRP-levels elevated in subjects with inflammation. In contrast, albumin considered a “late” negative acute phase protein [65] declines in conditions of inflammation [66] further indicating that in dogs suffering from CAD subclinical inflammation is present.

Our CAD cohort also had depleted iron-stores as indicated by the significantly lower levels of hepcidin and ceruloplasmin found in the circulation, while contrary to our expectations serum iron and TSAT-levels were elevated.

Iron homeostasis is very complex, as the different markers behave differently under iron-replete and depleted conditions and whether inflammation is present or not.

Hepcidin is considered the master regulator for iron absorption with elevated level hindering iron absorption and decreased levels facilitating iron absorption [30, 67]. Moreover, while upon inflammation elevated levels of hepcidin has been reported, this only seem to be true in subjects with adequate iron stores, as in anemic subjects with inflammation, hepcidin-levels have been repeatedly reported to be decreased [68–70].

The copper-containing ceruloplasmin is a ferroxidase that is elevated in the acute phase, but decreased in the chronic phase when iron stores are empty [71]. Its main function is to mobilize iron from the tissue (rather than from the gut) into the circulation. However when iron stores are depleted – even when anemia is still not present – ceruloplasmin-levels are lower than in subjects with sufficient iron [72].

We give evidence that in the CAD patients the iron-stores were depleted as indicated by significantly lower ceruloplasmin-levels. CAD patients seem to try to increase dietary iron uptake by decreasing hepcidin in the circulation to absorb more iron. Indeed, iron-deficiency in dogs has already been described to decrease hepcidin-levels [73]. In humans, hepcidin though also acting as positive acute phase protein in iron-repleted states, declines -even in inflammatory conditions-, when the iron stores are empty [68–70]. Also, in dogs with chronic kidney disease and thus inflammation, hepcidin-levels have been shown to be significantly lower in the presence of anemia [74].

Importantly, low iron stores are associated with symptoms regardless of the presence of typical microcytic, hypochromic anemia and is very difficult to recognize in patients with concurrent (subclinical) inflammation [75]. Here similarly, we associated clinical symptoms PVAS/Cadesi with ceruloplasmin and a lower iron status, but could not establish a direct dependency with the symptom burden. However, in CAD but not the healthy cohort, inflammation-markers (CRP, albumin) were associated with serum iron, PCV/hematocrit-values and neutrophils, which was not seen in the healthy cohort, in which serum iron seemed to be dependent only on hepcidin.

In the diagnostic iron panel also the total iron binding capacity (TIBC) is measured, and represent the maximum concentration of iron that can be bound in plasma. However TIBC is often of limited clinical value in small animals, as true serum or tissue iron levels are not assessed and because TIBC values do not change dramatically in disease [27]. In dogs, transferrin saturation (in contrast to humans, in which transferrin is directly measured), is measured indirectly via TIBC.

Iron-deficiency develops slowly, with initially result in an increase in reticulocyte production, and only when iron stores are depleted, the absolute count of red blood cells decrease. Importantly, due to the lack of heme and to the associated reduced hemoglobin synthesis, the red blood cells become more fragile. Consequently, this may result in mild hemolysis aggravating iron-deficiency, but also affecting measurements of serum iron-levels.

Indeed, we interpret the elevated serum iron and TSAT-levels in our CAD patients due to mild hemolysis that affected our measurements. A subsequent literature search confirmed that interpretation of serum iron laboratory data is complicated due to the strong circadian rhythm of iron and that elevated levels may be a result of hemolysis or prior food consumption [27, 76]. Further, our sample collection included non-fasting blood and as such post-prandial elevation of serum iron cannot be excluded. Moreover, many immunosuppressants such as corticosteroids are known to increase serum iron levels by incompletely understood mechanisms [28].

Thus, we show here for the first time, that well-nurtured, well-treated, non-anemic dogs with atopic dermatitis have iron-deficiency. Our CAD-cohort showed all signs of iron-deficiency (without anemia), in which their iron stores were depleted and which was accompanied by the presence subclinical inflammation as evidenced by higher levels of positive acute phase proteins (total protein, CRP) and lower levels of negative acute phase proteins (albumin) [65, 66].

Iron-deficiency develops slowly with the lower content of hemoglobin, also causing that the red blood cells are more fragile and more prone to hemolysis. This may also explain the higher serum iron-levels detected in our hands in the CAD-cohort, but may also be due to the immunosuppressive medication (Lokivetmab [77], ciclosporin [78], glucocorticoids [79], which all affect the iron-status.

Whether iron-deficiency is the cause or the consequence remains to be determined as both seemed to be true. A chronic lack in dietary iron will result in immune activation as the major cell recycling and distributing iron in mammals are macrophages, which shift towards a more inflammatory phenotype when they are unable to provide the tissues with sufficient iron [30]. In contrast, a high iron-turnover is a characteristic of regulatory macrophages, which can only occur under iron-sufficient conditions. Hence, in apparently healthy iron-deficient individuals subclinical inflammation is already present [62–64]. On the other hand, once inflammation is present, iron recycling is severely hampered as inflammatory macrophages do not distribute iron, and dietary uptake is impeded (mucosal block). Consequently, the risk of becoming anemic-despite adequate nutrition- nearly doubles within a year in children with atopic dermatitis [2] and increased 4 fold in patients with asthma within 5 years [80]. Similar scenarios have to be expected in canine patients. On the one hand nutritional deficiencies may exacerbate inflammation, but also due to malabsorption in chronic inflammatory conditions the risk of iron-deficiency – despite presumably adequate nutrition- is very prevalent.

## Conclusion

In conclusion, our study reveals that in well-nourished dogs with atopic dermatitis, the iron stores are depleted and subclinical inflammation is present.

## Methods

### Animals and sample collection

Samples of two study populations were compared in this study: 94 plasma samples from adult client-owned healthy dogs were used to compose a control group. Data and blood samples originated from a behavioural study carried out by the Domestication Lab at the University of Veterinary Medicine that took place at the same time. In that project, all methods were carried out in according to Austrian guidelines and regulations, and approval was given by the Ethics Committee of the University of Veterinary Medicine Vienna and the Austrian Federal Ministry of Science and Research (Ref: BMBWF 20221 − 0.210.26). Heparin-plasma samples were centrifuged at 3000 rpm for 3 min directly after collection and stored frozen at -20^o^C until analysis. Animals over 1 year of age and 5 kg were considered after a diagnostic evaluation that included thorough history, physical exam, complete blood count (CBC), and plasma biochemistry analysis. Dogs that had received any medical treatment within 4 weeks prior to blood collection were excluded. Dogs in the healthy control group at the time of blood collection were fed various commercial food, either dry or canned, as diet was not standardized. However, dogs with known food allergy or food responsive enteropathy were excluded. All blood samples were collected between 7:00 and 12:00, and all animals were fasting for at least 8 h prior to the blood collection. Dogs with CRP-levels over 10 µg/l were considered having subclinical low-grade inflammation and were excluded from the healthy control cohort.

For the CAD cohort, we used 34 surplus blood serum samples that were collected in the Dermatology Service of the Small Animal Clinic of the University of Veterinary Medicine Vienna, and all animals had a confirmed diagnosis of CAD, in compliance to current guidelines(1). Severity of disease in dogs with CAD was assessed with the Canine Atopic Dermatitis Extent and Severity Index 4 (CADESI-4).(2) The severity of pruritus was assessed with the Pruritus Visual Analog Scale (PVAS), a simple scoring system, in which pruritus is graded from 1 to 10 with increasing severity (3, 4). Patients with other diseases than atopic dermatitis were not included, atopic dogs with skin or ear infections secondary to CAD were included. Patients on systemic glucocorticoids or oclacitinib were not included in the study.

### Routine bloodwork

An automated complete blood cell count was performed using the hematology analyzer ADVIA 2120i (Siemens Healthcare Diagnostics GmbH, Vienna, Austria). Blood smears were prepared and stained by an automated stainer using a modified Wright stain. Numerical changes in total leukocyte counts, neutrophils, and lymphocytes exceeding 25% of the upper or the lower reference limit of the respective cell population and/or scatterplots indicating inappropriate cell separation prompted a microscopic slide evaluation by a senior technician to confirm abnormalities present in automatic counts, and if necessary, a microscopic count of 100 cells was carried out. Plasma or serum biochemistry analysis was carried out with the Cobas c 501 (Roche Diagnostics, Austria).

### ELISA measurements of plasma and serum ferritin, CRP, transferrin and hepcidin

Serum or plasma canine ferritin was measured according to manufacturer instructions by a competitive ELISA (BlueGene E08F0136 Canine Ferritin Heavy Polypeptide ELISA, Shanghai, China) with serum samples diluted 1:4 for analysis.

High sensitivity C-Reactive Protein (Hs-CRP) was measured by a sandwich-ELISA against Dog C Reactive Protein ELISA kit (Abcam ab157698, Cambridge UK), canine serum/plasma transferrin with Abcam’s canine transferrin ELISA kit (ab157704) and for hepcidin with Duoset human hepcidin ELISA (due to the high cross-reactivity, R&D Systems DY8307, Minnneapolis, MN) according to the manufacturers’ instruction. Samples for hsCRP-ELISA were diluted 1:4, for transferrin 1:50.000 and for hepcidin 1:150. Between each step, rigorous washing was performed, before measuring absorbance at 450 nm.

### Ceruloplasmin activity

Ceruloplasmin activity [81, 82] was measurement with N, N-dimethyl-p-phenylenediamine dihydrochloride (DMPD, Sigma D4139, St. Louis, Missouri), a compound that produces a long-lived radical cation, as described by Verde at al 2002 [83]. To 100 µl 0.1 M acetate buffer, pH 4.8/well, 10 µl DMPD/well and 2 µl sample/standard (0-400 nM iron with 3% hydrogenperoxide) was added. Colorimetric reactions were monitored at 505 nm every 5 min for 60 min. The oxidative activity was calculated based on the standard row and expressed in IU [84].

### Serum iron, total TIBC and unsaturated iron binding capacity UIBC, TSAT

Serum iron, TIBC and UIBC was measured according to the method described by Yamashita et al. [85]. For serum iron measurements, serum or plasma samples were diluted 1 + 1 in 0.89% NaCl, whereas for UIBC-analysis samples were 1 + 1 diluted with neutral 62 µmol/L ferrous-solution in 75 mmol/L sodium hydrogen carbonate/ 375 mmol/L TRIS buffer, pH 8.4. Briefly, 30 µl of serum iron sample/UIBC sample/standards (for iron and copper) were mixed with 100 µl Nitro-Paps (Dojindo N031, Mashiki, Tabaru, Japan) color reagent (125mM Nitropaps, 3% SDS, 45mM ascorbic acid, 0.3 M acetate buffer pH 5) and absorbance was measured at 592 nm after 10 min to obtain serum iron and copper levels. Subsequently, 10 µl of 0.4 M thioglycolic acid in 50mM sodium hydroxid was added (to remove copper from Nitropaps) incubated for 5 min and a 2nd measurement was performed at 592 nm.

The total iron binding capacity (TIBC) was calculated through summation of serum iron to UIBC as serum iron + UIBC = TIBC. Transferrinsaturation TSAT was calculated as serum iron/ TIBC in % using following equation serum iron/TIBC*100.

### Statistical analysis

The statistical analysis was performed with GraphPad Prism 10. Data were first tested for normality with the with Anderson-Darling-test. Parametric data were compared with Student’s t-test, not normally distributed, nonparametric red and white blood cell parameter were compared with Mann- Whitney U test to detect a shift in the median, non-parametric humoral parameters were with Kolmogorov-Smirnov-test to detect differences in the shape of the distribution. Non-parametric Spearman r correlation was used to detect associations and simple linear regression were performed for testing for dependency of variables. A *p* < 0.05 was considered significant.

## Data Availability

All data supporting the findings of this study are available within the paper. The datasets analysed for the current study are available from the corresponding author on reasonable request.

## Abbreviations

CAD: Canine atopic dermatitis
CADESI-4: Canine Atopic Dermatitis Extent and Severity Index
CRP: C-reactive protein
MCH: Mean corpuscular hemoglobin
MCV: Mean corpuscular volume
PCV: Packed cell volume
PVAS: Pruritis visual analogue scale
TIBC: Total iron binding capacity
TSAT: Transferrin saturation
